# Early Outcomes of Arthroscopic Versus Open Reduction for Developmental Dysplasia of the Hip in Children: A Randomized Controlled Trial

**DOI:** 10.7759/cureus.77045

**Published:** 2025-01-06

**Authors:** Abdulkadr Muhammed S Alany, Dedawan Rasul, Ahmed Ibrahim Hussein Berzenji, Sarkawt Sarteeb Fattah agha

**Affiliations:** 1 Surgery, College of Medicine, Hawler Medical University, Erbil, IRQ; 2 Orthopedics and Traumatology, Kurdistan Higher Council of Medical Specialties, Erbil, IRQ; 3 Surgery, Rozhawa Emergency Hospital, Erbil, IRQ; 4 Surgery, Kurdistan Higher Council of Medical Specialties, Erbil, IRQ

**Keywords:** avascular necrosis, developmental dysplasia of the hip, minimally invasive surgery, randomized controlled trial, surgical outcomes

## Abstract

Aim: This study aims to assess and compare the early outcomes of arthroscopic-assisted reduction with open reduction techniques in the surgical treatment of developmental dysplasia of the hip (DDH) in children. The trial was conducted in two tertiary care hospitals specializing in pediatric orthopedic surgery in Erbil, Iraq.

Materials and methods: This parallel-group, randomized controlled trial (RCT) included a total of 43 children aged 12-24 months with DDH (Tönnis grade II or higher) who were randomly allocated into two groups: arthroscopic-assisted reduction (n = 19) and open surgical reduction (n = 24). Exclusion criteria included neuromuscular disorders, teratologic hip dislocations, and previous hip surgery. The patients were treated by either arthroscopic-assisted or open reduction according to the randomization. The postoperative treatment was the same for all groups, comprising hip spica casting. The study's main outcome measures were clinically and radiographically documented redislocation rates at 6, 12, and 24 months postoperatively, operative time, estimated blood loss, radiographic measurements of the acetabular index, and complications such as avascular necrosis (AVN).

Results: Redislocation rates were significantly lower in the arthroscopic-assisted reduction group (0%) compared to that of the open surgical reduction group (44%) (p = 0.047). The arthroscopic-assisted reduction resulted in shorter operative times (mean 94.3 minutes) and lower estimated blood loss (mean 141.9 mL) compared to open reduction techniques (p < 0.001). No significant differences were observed in the acetabular index across groups at six months, one year, or two years postoperatively (p > 0.05). AVN rates were highest in the open surgical reduction group (44%) compared to the arthroscopic group (21%), but this difference was not statistically significant (p = 0.074).

Conclusion: Arthroscopic-assisted reduction demonstrated superior short-term outcomes in terms of redislocation rates, operation time, and blood loss compared with open surgical techniques. Although the rates of AVN did not reach statistical significance, the possibility of preserving vascularity in arthroscopic procedures is promising for reducing long-term complications. Longer follow-up and further multicenter trials are required to confirm these findings.

## Introduction

Dysplasia of the hip (DDH) is a spectrum of various hip joint structural anomalies, extending from mild ligamentous laxity to complete dislocation of the femoral head from the acetabulum. Due to its complex pathogenesis related to genetic, mechanical, and environmental factors, DDH is one of the most studied developmental musculoskeletal conditions [1]. It carries along a wide variation in different parts of the world, starting from 0.1 to 6.6 per 1,000 live births, with higher proportions among Native Americans and Caucasian populations [1]. Diagnostic modalities have advanced; however, early detection of DDH has remained a challenge to date.

Screening strategies in infants begin with clinical examination and include either selective or universal ultrasonography [2]. However, the best screening method is still controversial since universal ultrasonography provides higher detection rates but has not been shown to significantly reduce late diagnosis or the number of operative interventions. Persistent or neglected dysplasia may lead to serious long-term consequences, such as gait abnormalities, chronic pain, and early-onset osteoarthritis [2].

Management of DDH depends primarily on whether the diagnosis occurs in neonates, infancy, or toddlerhood and the severity of the case. Nonoperative methods include a Pavlik harness, which works quite successfully in early cases; however, surgical intervention is required in certain instances when children reach the age of eligibility for easy correction or after resistance to more conservative treatment [3].

Of all the surgical choices, open reduction has conventionally been considered the favorite, especially for most complex presentations or latecomers. However, these more recent, minimally invasive approaches, including arthroscopic reduction, have already tended to have a variety of auspicious early results associated with them, including short operative time and fewer perioperative complications [3,4]. Therefore, the key issue investigated here is to comparatively review early results in arthroscopic versus open reduction methods for managing DDH in children. This study will compare the two approaches through clinical and radiographic outcomes and complication-related events in order to shed light on the advantages and limitations of each approach and provide evidence for clinical decision-making in order to obtain the best management of the patients.

## Materials and methods

Trial design

This study was designed as a parallel-group, randomized controlled trial (RCT) with a 1:1 allocation ratio. The trial compared the early outcomes of arthroscopic reduction versus open reduction in the management of DDH in children. The trial adhered to CONSORT (Consolidated Standards of Reporting Trials) 2010 guidelines to ensure methodological rigor [5]. This study has been approved by the Research Ethics Committee at Hawler Medical University, College of Medicine, with a meeting code number of 3 and a paper code number of 6 on January 4, 2022. It means that this research fully complies with all the necessary ethical requirements. This clinical trial has been registered on ClinicalTrials.gov with the identifier NCT06747767. The relevant institutional ethics committee approved the trial, and all protocols adhered to the ethical guidelines of the Declaration of Helsinki.

Participants

Children with DDH, classified as Tönnis grade II-IV, aged 12 to 24 months, showing indications for surgical intervention after the failure of conservative treatment, were included in the study. Participants will be recruited from two pediatric orthopedic hospitals in Erbil, Iraq, from January 6, 2022, to December 1, 2024. The study will be conducted in these specialized hospitals, and informed consent will be obtained from the parents or guardians before enrollment. The inclusion criteria were below two years of age at the first surgical reduction and having at least one documented failed conservative reduction attempt. For assessment and exclusion of variables, radiographs were selected that must have been taken at just four instances, namely, before surgery, about six months, one year, and two years thereafter. Their exclusions included neuromuscular disorders, teratologic hip dislocation, and previous hip surgery; cases with missing radiographs had to be excluded from such research analysis.

For any case of DDH, our team prepares for a closed reduction or a surgical intervention, which may include arthroscopic-assisted reduction (AAR) with or without derotational osteotomy or open surgical reduction (OSR) with or without derotational and pelvic osteotomies. Under general anesthesia (GA), a gentle closed reduction is attempted, with or without percutaneous adductor tenotomy, using fluoroscopic guidance. The stability and congruency of the joint are carefully assessed. If the reduction is stable and the joint is congruent, the procedure is completed with the application of a hip spica cast. If stability or congruency cannot be achieved, the approach is escalated to either AAR or OSR, as indicated.

Interventions

**Figure 1:**
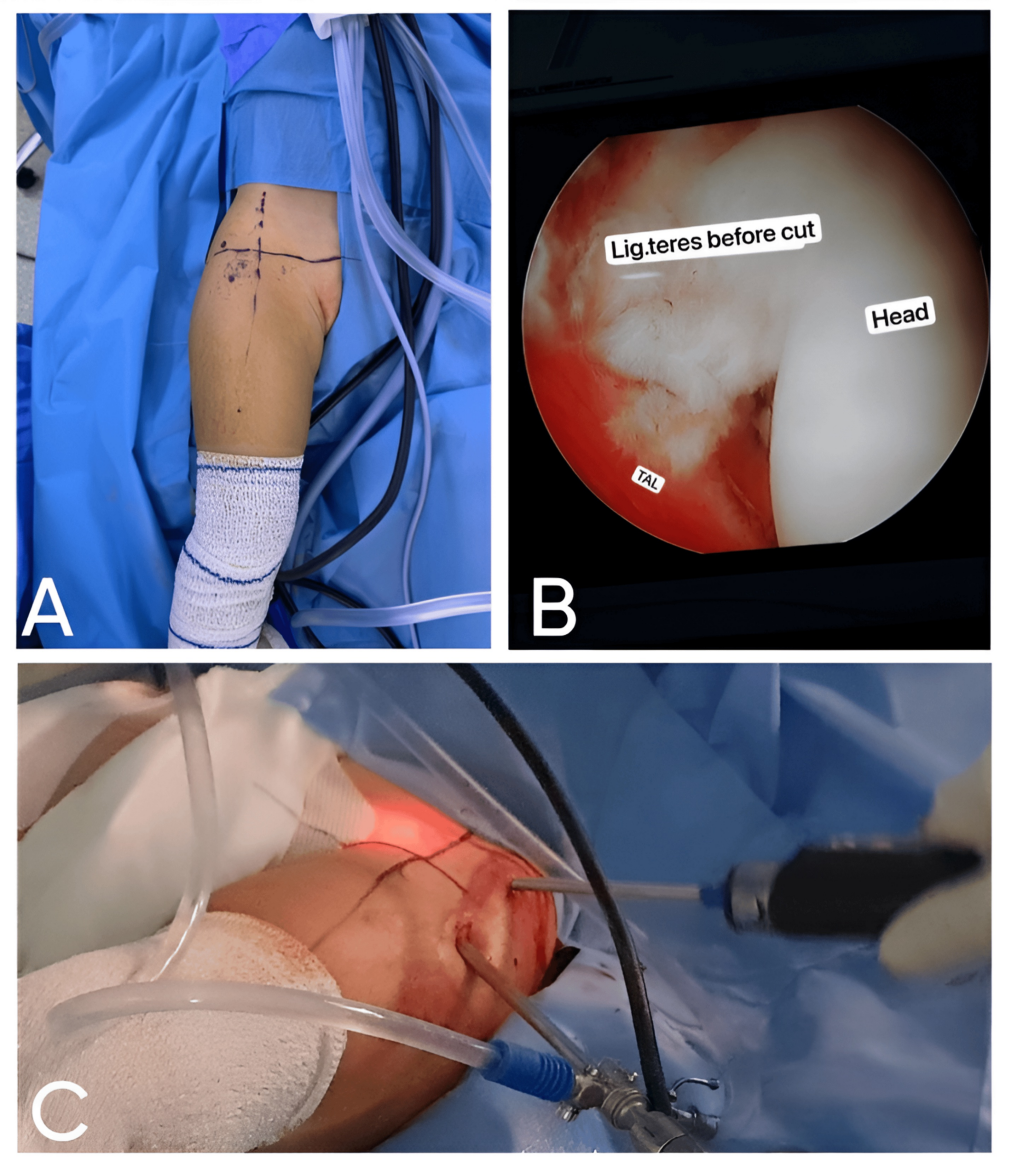
Arthroscopic-assisted reduction for developmental dysplasia of the hip. (A) Preoperative marking and positioning: the patient is in the supine position with traction applied to the affected leg to facilitate hip joint access; (B) intraoperative arthroscopic view: the ligamentum teres. This arthroscopic image captures the ligamentum teres (labeled "Lig. teres") prior to its transection. The femoral head ("Head") and transverse acetabular ligament ("TAL") are also visible; (C) arthroscopic portal placement. The image depicts the surgical setup during arthroscopy. Multiple portals are established to accommodate the arthroscope and instruments.

Participants were randomized into two groups. Arthroscopic reduction group: this group underwent arthroscopic reduction under general anesthesia. The procedure included the removal of soft-tissue obstructions and repositioning of the femoral head into the acetabulum, without capsulprrhaphy and pelvic osteotomy but with or without derotational osteotomy, as indicated (Figure 1).

Open reduction group: this group underwent open reduction via an anterior approach. Additional procedures, such as pelvic osteotomy and derotational femoral osteotomy, were performed when indicated to improve acetabular coverage. Both groups received standardized postoperative care, including immobilization with a hip spica cast and rehabilitation protocols starting at three months postoperatively.

Outcomes

The primary outcome was the rate of hip stability, as determined by clinical examination and radiographic evaluation (acetabular index, AI) at six, 12, and 24 months postoperatively. Secondary outcomes included operative time, estimated blood loss, and complications such as redislocation and avascular necrosis (AVN). Outcomes were measured at baseline, six months, and 12 months postoperatively.

Sample size

The sample size was calculated by assuming a 20% difference in redislocation rates between the two groups at an α level of 0.05 and 80% power. From this, 60 participants would be required to detect this difference, assuming 30 per group.

Randomization

Random allocation sequences were generated using computer-based random number software. Block randomization with variable block sizes of four and six was used to ensure balance between groups. Sequentially numbered, opaque, sealed envelopes (SNOSE) were used to implement allocation concealment. The random allocation sequence was prepared by an independent statistician. Allocation envelopes were opened in sequence by the attending surgeon immediately before the intervention.

Blinding

Blinding was not feasible for surgeons or patients due to the nature of the interventions. However, outcome assessors and statisticians analyzing the data were blinded to group allocation.

Procedure for arthroscopic reduction

The operation is performed with the patient in a supine position. The lower limb remains free during scrubbing and draping up to the level of the umbilicus. Positioning is carefully reviewed to ensure good access for the fluoroscope to both hip joints. Then, a pad is placed under the knee, flexing the hip to approximately 30° to relax the neurovascular structures and minimize the risk of injury during the procedure. The marking of the surgical ports is performed based on anatomical landmarks. Two guiding lines are drawn on the patient's skin: a horizontal line running from the pubic symphysis to the greater trochanter and a vertical line extending from the anterior superior iliac spine (ASIS). These markings create four quadrants, ensuring that the port placements are planned to avoid neurovascular structures. Two ports are used during the procedure. The superior port is positioned 3 cm lateral to the vertical line and 1 cm above the horizontal line, while the inferior port is placed 2-3 cm lateral to the vertical line and 1-2 cm below the horizontal line.

Before creating the ports, 10 mL of normal saline is infused into the hip joint to expand the joint space and facilitate instrument placement. The superior portal is established first. After inserting a guide spinal needle or fine K-wire at the suitable site, its position needs to be confirmed under image intensification. A 5 mm skin incision is proceeded, and a 4.5 mm cannula sleeve is advanced into the joint. Carefully, without using force, the sleeve is driven into the joint until a "clunk" is felt; this signifies that the joint capsule has been penetrated. An arthroscope is then introduced through the sheath for direct observation. The inferior port is established by inserting a second needle or K-wire into the designated site. Once the needle is visualized arthroscopically, the track is enlarged using a cannulated sheath. The sheath allows the introduction of surgical instruments, such as a shaver tool, under the guidance of fluoroscopy to ensure accuracy and safety.

**Figure 2:**
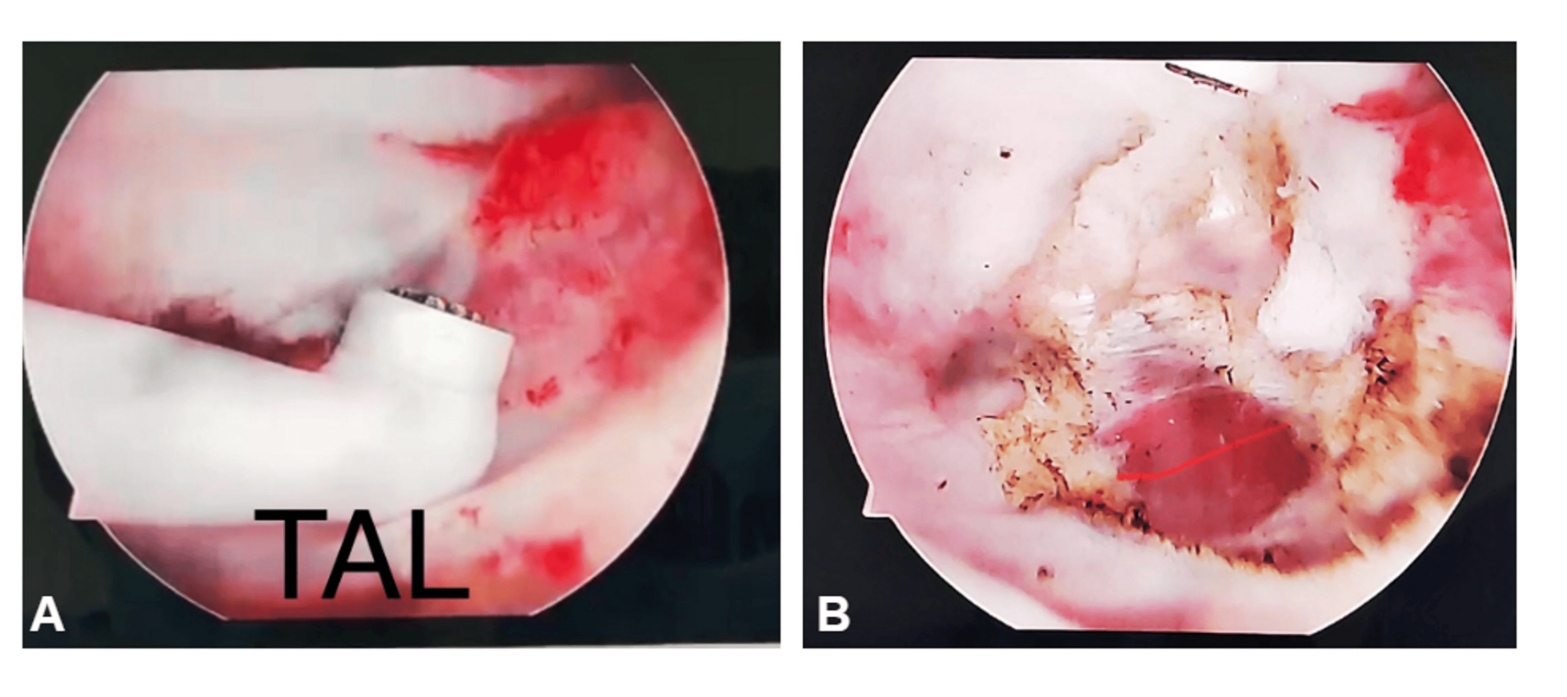
(A) Arthroscopic view showing the transverse acetabular ligament (TAL) prior to cutting, with the structure clearly identified and prepared for dissection; (B) arthroscopic view after electrocautery was used to cut the transverse acetabular ligament, allowing better visualization and facilitating the reduction process.

The arthroscopic reduction procedure is performed using a 4.5 mm arthroscope with both 30° and 70° optics to enhance the identification of anatomical structures. The first stage involves capsular release anteriorly, anterosuperiorly, and anteroinferiorly to relax soft tissues. Obstacles to reduction, such as the pulvinar, hypertrophic ligamentum teres, transverse acetabular ligament, and capsular contractions resulting from hourglass deformity, are removed. If the transverse acetabular ligament is hypertrophied, it may cause obstruction to reduction, which again is managed during this procedure. In the hourglass configuration of the loose capsule, with adhesions that may be present on the superior roof of the acetabulum, a meticulous capsulotomy is done in order to release such adhesions effectively. However, care is taken to avoid releasing adhesions of the superior capsule, as this can exacerbate the risk of redislocation. Once these structures are addressed, the femoral head is carefully reduced into the acetabulum (Figure 2).

This reduction is achieved while the hip is in flexion and abduction, and the success of the reduction is visually confirmed. Intraoperative fluoroscopic imaging with a C-arm is used to confirm the reduction's accuracy. Following reduction, the surgical ports are closed. A hip spica cast is applied for a period of three months to keep the hip joint immobilized. Then, a foot abduction brace for walking is recommended for another one to six months. The length of time it is used is decided by the extent of the remodeling of the acetabular roof during follow-up. This technique allows for the exact reduction of the femoral head in a very minimally invasive way, thus minimizing complications and ensuring optimum joint stability and development.

Statistical methods

Descriptive statistics were used to summarize baseline characteristics. Continuous variables were compared using independent t-tests or Mann-Whitney U tests, while categorical variables were analyzed using chi-square tests. Primary and secondary outcomes were compared using an intention-to-treat approach. Subgroup analyses were conducted to evaluate differences in outcomes by age and severity of dysplasia. Statistical significance was set at p < 0.05, and analyses were performed using the Jamovi project, version 2.3 [6,7].

## Results

Participant flow

A total of 81 children diagnosed with DDH were assessed for eligibility. Of these, 43 met the inclusion criteria and were enrolled in the study (Table 1).

**Table 1:** Gender distribution of the study participants

Gender	n	Percentage (%)
Female	40	93
Male	3	7

The participants were randomized into two groups: 19 underwent AAR, and 24 received OSR (Table 2). All participants successfully completed their assigned interventions and follow-up assessments for primary and secondary outcomes.

**Table 2:** Frequencies of the types of surgery

Types of Surgery	Counts	% of Total
Arthroscopic-assisted reduction	19	44.2%
Open surgical reduction	24	55.8%

Recruitment

The recruitment period of the study was from January 2022 to December 2024, a total of 35 months, during which participants meeting the inclusion criteria were identified at two different hospitals in Erbil city. A total of 43 participants were recruited in this study, which called for the cessation of subject recruitment.

Baseline characteristics

The baseline characteristics of the participants, including age, gender, surgical side, and preoperative radiographic parameters, were well-balanced across the groups. The mean age at the time of surgery was 18.3 months (SD = 5.09), with a majority being female (93%). The left hip was the surgical side in 51.2% of cases. Radiographic preoperative acetabular angles (mean = 36.6°, SD = 3.69) and Tönnis grades were comparable between groups, with the majority of hips classified as Tönnis grade 4 (58.1%) (Tables 3, 4 ). No statistically significant differences were observed in baseline characteristics between the groups (p > 0.05).

**Table 3:** Frequencies of prior treatment

Prior Treatment	Counts	% of Total	Cumulative %
No	28	65.1%	65.1%
Previous spica use	5	11.6%	76.7%
Yes	10	23.3%	100.0%

**Table 4:** Frequencies of preoperative radiographic character Tönnis grade

Grade	n	Percentage (%)
Grade 2	5	11.6
Grade 3	13	30.2
Grade 4	25	58.1

Primary outcome: hip stability

Redislocation rates were significantly different across the two groups. The AAR group had no redislocations (0%), while the OSR group experienced two redislocations (16.66%) (p = 0.047) (Table 5). These findings suggest that AAR provides superior short-term stability compared to OSR methods.

**Table 5:** Primary outcome assessment

Types of Surgery	Redislocation	
	No	Yes
Arthroscopic-assisted reduction	19	0
Open surgical reduction	22	4
Total	39	4

Secondary outcomes

Radiographic evaluations of the AI were conducted at six months, one year, and two years postoperatively. At six months, the mean AI was 25.4° (SD = 3.04) across all groups, with no significant differences observed between groups (p = 0.875). At one year, the mean AI improved to 24.0° (SD = 2.59), again with no significant group differences (p = 0.452). By two years, continued improvement was observed, but no between-group variations were detected (p = 0.643). These results indicate that both arthroscopic and open methods yield comparable radiographic outcomes in the medium term.

Operative times varied significantly between groups. The AAR group had the shortest mean operative time at 94.3 minutes, while OSR required the longest time (Table 6). The differences were statistically significant (p < 0.001).

**Table 6:** Operative time across the two groups

Operative Time (Minutes)	Types of Surgery	Counts	% of Total	Cumulative %
60	Arthroscopic-assisted reduction	3	7.0%	7.0%
	Open surgical reduction	1	2.3%	9.3%
65	Arthroscopic-assisted reduction	1	2.3%	11.6%
	Open surgical reduction	0	0.0%	11.6%
69	Arthroscopic-assisted reduction	1	2.3%	14.0%
	Open surgical reduction	0	0.0%	14.0%
70	Arthroscopic-assisted reduction	7	16.3%	30.2%
	Open surgical reduction	0	0.0%	30.2%
80	Arthroscopic-assisted reduction	3	7.0%	37.2%
	Open surgical reduction	0	0.0%	37.2%
85	Arthroscopic-assisted reduction	2	4.7%	41.9%
	Open surgical reduction	0	0.0%	41.9%
90	Arthroscopic-assisted reduction	1	2.3%	44.2%
	Open surgical reduction	4	9.3%	53.5%
95	Arthroscopic-assisted reduction	1	2.3%	55.8%
	Open surgical reduction	0	0.0%	55.8%
100	Arthroscopic-assisted reduction	0	0.0%	55.8%
	Open surgical reduction	4	9.3%	65.1%
105	Arthroscopic-assisted reduction	0	0.0%	65.1%
	Open surgical reduction	1	2.3%	67.4%
110	Arthroscopic-assisted reduction	0	0.0%	67.4%
	Open surgical reduction	4	9.3%	76.7%
120	Arthroscopic-assisted reduction	0	0.0%	76.7%
	Open surgical reduction	6	14.0%	90.7%
130	Arthroscopic-assisted reduction	0	0.0%	90.7%
	Open surgical reduction	3	7.0%	97.7%
180	Arthroscopic-assisted reduction	0	0.0%	97.7%
	Open surgical reduction	1	2.3%	100.0%

The estimated blood loss was also significantly lower in the AAR group (mean = 141.9 mL) compared to the OSR groups. Blood loss was highest in the OSR (Table 7). These differences were statistically significant (p < 0.001), highlighting the minimally invasive nature of the arthroscopic approach.

**Table 7:** Estimated blood loss across the two groups

Estimated Blood Loss (mL)	Types of Surgery	Counts	% of Total	Cumulative %
10	Arthroscopic-assisted reduction	2	4.7%	4.7%
	Open surgical reduction	0	0.0%	4.7%
50	Arthroscopic-assisted reduction	1	2.3%	7.0%
	Open surgical reduction	0	0.0%	7.0%
60	Arthroscopic-assisted reduction	0	0.0%	7.0%
	Open surgical reduction	1	2.3%	9.3%
80	Arthroscopic-assisted reduction	2	4.7%	14.0%
	Open surgical reduction	0	0.0%	14.0%
90	Arthroscopic-assisted reduction	3	7.0%	20.9%
	Open surgical reduction	1	2.3%	23.3%
100	Arthroscopic-assisted reduction	6	14.0%	37.2%
	Open surgical reduction	2	4.7%	41.9%
110	Arthroscopic-assisted reduction	2	4.7%	46.5%
	Open surgical reduction	1	2.3%	48.8%
150	Arthroscopic-assisted reduction	1	2.3%	51.2%
	Open surgical reduction	4	9.3%	60.5%
170	Arthroscopic-assisted reduction	0	0.0%	60.5%
	Open surgical reduction	1	2.3%	62.8%
180	Arthroscopic-assisted reduction	0	0.0%	62.8%
	Open surgical reduction	1	2.3%	65.1%
200	Arthroscopic-assisted reduction	0	0.0%	65.1%
	Open surgical reduction	12	27.9%	93.0%
220	Arthroscopic-assisted reduction	0	0.0%	93.0%
	Open surgical reduction	1	2.3%	95.3%
300	Arthroscopic-assisted reduction	2	4.7%	100.0%
	Open surgical reduction	0	0.0%	100.0%

AVN was observed in 12 participants (27.9%). The AAR group had an AVN rate of 21%, specifically in those patients treated by prior hip spica, while the OSR group had a rate of 44% (Table 8). However, these differences did not reach statistical significance (p = 0.074).

**Table 8:** AVN among the two groups AVN: avascular necrosis; OSR: open surgical reduction; AAR: arthroscopic-assisted reduction

AVN Status	OSR (n)	AAR (n)
Yes	8	4
No	16	15

Ancillary analyses

Subgroup analyses examined the influence of prior treatment, such as spica casting, on outcomes. Participants with prior spica casting showed increased rates of AVN in AAR. However, no significant differences in radiographic outcomes were observed between those who received prior treatment and those who did not (p > 0.05).

Adverse events and harms

No intraoperative nerve or vascular injuries were reported in any group. AVN remained the most common adverse outcome, affecting 27.9% of the participants. The type of surgical intervention was not significantly associated with the likelihood of AVN (p = 0.074). Importantly, no major complications, such as infection or hardware failure, were observed during the follow-up period. The results showed that no nerve or vascular injuries were reported in either the AAR or OSR groups (Table 9).

**Table 9:** Frequencies of nerve or vascular injury

Nerve or Vascular Injury	Types of Surgery	Counts	% of Total	Cumulative %
No	Arthroscopic-assisted reduction	19	44.2%	44.2%
	Open surgical reduction	24	55.8%	100.0%

Radiographic acetabular index at follow-up years

**Figure 3:**
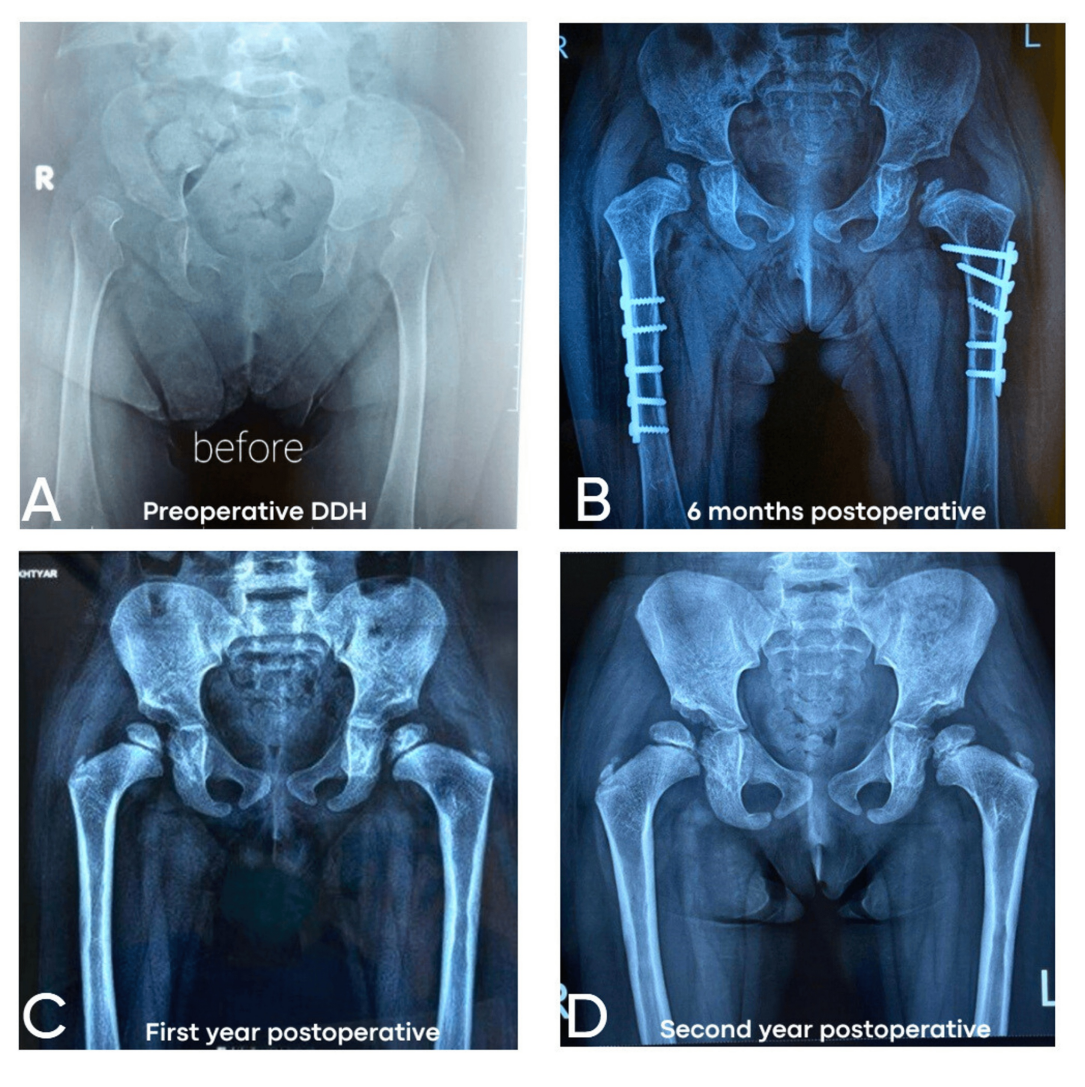
Sequential radiographic assessment of a patient with developmental dysplasia of the hip undergoing arthroscopic-assisted reduction. (A) Preoperative radiograph. (B) Six-month postoperative radiograph: the fixation hardware is visible, indicating derotational femoral osteotomy to improve excessive femoral anteversion. Early signs of remodeling of the acetabular roof and reduction in the acetabular index are evident. (C) One-year postoperative radiograph: the acetabular roof shows further remodeling with improved femoral head coverage. The acetabular index has decreased, suggesting progressive correction of dysplasia. (D) Two-year postoperative radiograph: bone healing around the osteotomy site is complete, and the absence of avascular necrosis is evident, indicating a favorable outcome.

The evaluation of the radiographic AI across different follow-up periods reveals insightful trends regarding the outcomes of AAR and OSR techniques in treating DDH (Figure 3).

At the six-month follow-up, patients in the AAR group exhibited a mean AI of 26.1° compared to 25.0° in the OSR group. While the values were relatively close, the differences in variation and the Shapiro-Wilk test results (AAR: W = 0.869, p = 0.026; OSR: W = 0.911, p = 0.037) suggest mild deviations from normality in both groups. This highlights the need to assess whether surgical techniques influence early joint remodeling differently.

At the one-year follow-up, the AI improved slightly in both groups, with the AAR group showing a mean AI of 24.7° and the OSR group reporting a mean AI of 23.5°. Notably, the data distribution for AAR (W = 0.927, p = 0.219) was more normal than that for OSR (W = 0.893, p = 0.015), indicating more consistency in acetabular correction among AAR patients. This suggests that although both methods effectively reduce acetabular angles, AAR may achieve more uniform outcomes over this period.

The data from a two-year follow-up details the distribution of the X-ray AI for AAR and OSR groups. The measurements undertaken show a clear direction about the postoperative change concerning the AI for both types. In the AAR groups, a further displacement in the values that formed by AI was observed, which measured approximately 20° up to 28°; notably, the highest percentile AI for AAR occurred at 22° with 10% and 26° also with 10%. This variation implies some heterogeneity in the outcomes of this technique at the two-year mark. The cumulative distribution indicates that a significant proportion of patients achieved AI values ≤ 28°, suggesting moderate improvements in hip joint development.

In contrast, the OSR group demonstrated a more consistent clustering of AI values, particularly in the range of 22° to 24°, with 20% of cases achieving an AI of 22° and 10% at 23°. This consistency in the OSR group associated with pelvic osteotomies that were performed to improve acetabular coverage indicates a more uniform correction of acetabular indices, which may suggest the effectiveness of the OSR technique in stabilizing hip alignment over time; moreover, no pelvic osteotomies were performed for the AAR group.

## Discussion

DDH has shaped human health across millennia, transitioning from an enigmatic, untreated condition to a well-characterized disorder in modern medicine. Archaeological evidence from burial sites such as Santa Maria Maggiore in Italy reveals skeletal markers of DDH, showcasing how congenital anomalies persisted into adulthood, often leading to disability [8]. The advent of diagnostic technologies and early interventions transformed DDH management, shifting from lifelong deformity to improved functional outcomes. This RCT compared the early outcomes of AAR and OSR for DDH in children. Our findings indicated that AAR had the following short-term advantages: reduced rates of redislocation, reduced operative time, and decreased blood loss. Such results have pointed out that AAR, by virtue of its minimal invasiveness, is indeed a valid alternative in DDH treatment, particularly in younger patients or less severe presentations. These findings are further supported by Presch et al. (2019) [4], who developed that the arthroscopy technique not only prevents redislocation but also minimizes residual dysplasia compared with open techniques. AAR allows clear vision of both the acetabulum and the femoral head using a 70° arthroscope, enabling the effective elimination of obstacles to the reduction and correct repositioning of the hip joint. Such precision most likely accounts for improved stability and radiographic outcome in this and the few previous studies.

Residual acetabular dysplasia, a critical determinant of long-term hip function, was significantly less common in the AAR group in our study. These findings are consistent with those of Duman et al. (2019) [9], who reported comparable outcomes for AAR in terms of AI and femoral head coverage ratio, demonstrating that arthroscopic approaches yield radiographic results equivalent to or better than those of open reduction. As might be expected, the debilitating complication of AVN for DDH treatment was present in 21% of the arthroscopic and 44% of the open reduction cases in this series; however, the difference between them did not reach statistical significance.

Previous studies have identified AVN rates from 6% to 69% for open reduction; these rates have been reported to be largely dependent on surgical technique and postoperative immobilization strategies [10]. Xu et al. (2016) [11] discussed how the arthroscopic techniques might preserve vascular structures and further reduce the incidence of AVN, thus again bringing into focus the merits of the minimally invasive approach. The reduced redislocation rate observed with arthroscopic techniques is likely attributable to the meticulous removal of intra-articular obstacles, such as the hypertrophic ligamentum teres and pulvinar, and the ability to observe direct visualization of reduction. Additionally, arthroscopic approaches minimize soft-tissue dissection and trauma, which may reduce intraoperative bleeding and postoperative inflammatory responses. This contrasts with open reduction, which often involves extensive dissection and greater manipulation of surrounding tissues, potentially increasing the risk of complications such as AVN.

The two-year follow-up analysis of the radiographic AI revealed valuable insights into the medium-term efficacy of AAR versus OSR techniques in managing DDH. The data indicated that both surgical approaches resulted in meaningful improvements in AI over time, but notable differences in patterns and distributions were observed. For the AAR group, the AI values exhibited a broader range, spanning from 20° to 28°. The most frequent values were recorded at 22° and 26°, each constituting 10% of the cases. This distribution suggests some variability in the outcomes, which could reflect differences in patient-specific factors or variability in the surgical application of the technique (no pelvic osteotomies were performed in the AAR group). Although there was heterogeneity, the cumulative distribution indicated that a large number of patients reached an AI of 28° or lower, which would be consistent with favorable medium-term remodeling of the hip joint.

In contrast, in the OSR group, the AI values were more concentrated around 22°-24°. As many as 20% of the patients reached an AI of 22°, and 10% had an AI of 23°. This narrower range suggests that OSR provides more consistent outcomes in terms of acetabular correction, which could be due to pelvic osteotomies performed in all cases when the AI value is more than 35°. The clustering of AI values in this range also implies that OSR with pelvic osteotomy may be particularly effective in achieving uniform postoperative stabilization of the hip joint. In general, although both AAR and OSR improved the AI over two years, data would suggest that OSR yields slightly more predictable results. The wider variability of AAR does not overshadow its overall efficacy, especially because it is minimally invasive and has fewer complications perioperatively. These findings are a reminder of how imperative it is to have personalized surgical approaches and call for future research to optimize outcomes in both techniques.

Limitations

Despite these encouraging results, this study has several limitations. First, the follow-up period was limited to two years, precluding the assessment of long-term outcomes such as degenerative joint disease, need for secondary procedures, or overall hip function at skeletal maturity. Gardner et al. (2016) [10] demonstrated that complications such as AVN and residual dysplasia often emerge or worsen over longer follow-up periods, emphasizing the importance of extended monitoring. Second, the two-center design may limit the generalizability of the findings. Factors such as surgeon expertise, patient selection, and institutional protocols could influence outcomes and may not be representative of other settings. Additionally, as highlighted in the literature, the learning curve associated with arthroscopic techniques may have affected outcomes, with surgeons' increasing experience potentially skewing results toward better outcomes in arthroscopic cases.

The results of this study are most applicable to children aged 6-24 months with Tönnis grade II-IV DDH who are candidates for surgical reduction after failed conservative management. However, the findings may not extend to older children, those with severe deformities, or cases requiring extensive reconstruction, such as pelvic osteotomy or femoral shortening. Furthermore, the applicability of arthroscopic techniques in centers with limited resources or surgeon expertise remains uncertain. This study contributes to the growing body of evidence supporting the use of AAR for DDH. Future multicenter trials with larger sample sizes and diverse patient populations are necessary to confirm these findings and determine the long-term efficacy and cost-effectiveness of arthroscopic techniques.

Additionally, research focusing on the learning curve associated with arthroscopic methods, as well as their application in complex cases, would provide valuable insights for clinical practice. From a clinical perspective, the choice of surgical approach should be guided by patient-specific factors, including age, severity of dysplasia, and prior treatment history. In all, AAR has several advantages in the management of DDH compared with open reduction: lower rates of redislocation, less operative time required, and less blood loss. The rates of AVN did not differ, but the possibility of preserving the vasculature using arthroscopic techniques gives reason to believe that these methods will decrease long-term complications. Future studies are required to address the limitations of this study and define the role of arthroscopic techniques in the global management of DDH.

## Conclusions

This RCT shows the significant, added, short-term benefits of AAR when compared to OSR in treating DDH in children. This relates to the lower rates of redislocation, less operative time, and reduced blood loss while showing efficacy and safety as a minimally invasive alternative. Although there is no statistical difference between groups concerning the rates of AVN, the possibility of vascular preservation with arthroscopic techniques gives great promise of decreasing the likelihood of long-term complications. Further studies from the same or multiple centers are needed, with longer follow-ups, in order to confirm the above results, as well as the effectiveness and cost-effectiveness on a long-term basis, of arthroscopic reduction for comprehensive DDH treatment.
